# Oral Adverse Events Associated with BRAF and MEK Inhibitors in Melanoma Treatment: A Narrative Literature Review

**DOI:** 10.3390/healthcare12010105

**Published:** 2024-01-02

**Authors:** Michele Basilicata, Vincenzo Terrano, Alessandro D’Aurelio, Giovanni Bruno, Teresa Troiani, Patrizio Bollero, Stefania Napolitano

**Affiliations:** 1 UOSD Special Care Dentistry, Department of Experimental Medicine and Surgery, University of Roma Tor Vergata, 00133 Rome, Italy; michele.basilicata@ptvonline.it (M.B.); daurelioalessandro@libero.it (A.D.); patrizio.bollero@ptvonline.it (P.B.); 2 UniCamillus-Saint Camillus, International University of Health Sciences, 00131 Rome, Italy; 3 Department of Precision Medicine, Università degli Studi della Campania “Luigi Vanvitelli”, 80138 Napoli, Italy; vincenzo.terrano@studenti.unicampania.it (V.T.); teresa.troiani@unicampania.it (T.T.); stefania.napolitano@unicampania.it (S.N.); 4 Department of Neuroscience, University of Padua, 35121 Padova, Italy; 5 Department of Industrial Engineering, University of Tor Vergata, 00133 Rome, Italy

**Keywords:** BRAF inhibitors, melanoma, MEK inhibitors, oral adverse events, special care dentistry

## Abstract

Background: Melanoma cancer represents the most lethal type of skin cancer originating from the malignant transformation of melanocyte cells. Almost 50% of melanomas show the activation of BRAF mutations. The identification and characterization of BRAF mutations led to the development of specific drugs that radically changed the therapeutic approach to melanoma. Methods: We conducted a narrative review of the literature according to a written protocol before conducting the study. This article is based on previously conducted studies. We identified articles by searching electronic databases (Medline, Google Scholar and PubMed). We used a combination of “melanoma”, “Braf-Mek inhibitors”, “ targeted therapy” and “oral side effects”. Results: Eighteen studies were reported in this article showing the relationship between the use of targeted therapy in melanoma cancer and the development of oral side effects, such as mucositis, hyperkeratosis and cellular proliferation. Conclusion: Targeted therapy plays an important role in the treatment of melanoma cancer, showing a notable increase in response rate, prolonged progression-free survival and overall survival in BRAF-mutated melanoma patients. Oral side effects represent a common finding over the course of treatment. However, these adverse effects can be easily managed in a multidisciplinary approach involving collaboration between medical oncologists and dental doctors.

## 1. Introduction

### 1.1. Melanoma Cancer

Melanoma is the deadliest form of skin cancer and is caused by the malignant transformation of melanocytes [1]. The incidence of melanoma is around 1.7% of all newly diagnosed primary malignant cancers, and its mortality rate is around 0.7% of all cancer mortality. Looking at the incidence and mortality of melanoma in different countries, the rate in Australia, New Zealand, Europe and North America is relatively high, while the rate in Africa is relatively low [2]. The age-standardized incidence rate is 3.8/100,000 for males and 3.0/100,000 for females, with cumulative lifetime risks of 0.42% and 0.33%, respectively [3].

Recent advances in drug development have improved the survival of patients affected by melanoma cancer. BRAF/MEK gene inhibitors and anti-PD1 antibodies, in particular, have totally revolutionized the management of this disease [2]. Activated BRAF mutation occurs in approximately 50% of cutaneous melanoma [4,5]. Actually, almost 300 BRAF mutations have been discovered, the most common being the V600E (valine to glutamic acid; 70–88%) [6,7,8]. The identification and characterization of BRAF mutations led to the development of specific drugs that radically changed the therapeutic approach to melanoma. These results led to the approval of BRAF plus MEK inhibitors for high risk resected (stage III) and advanced melanoma patients [9,10], underlining the importance of the early molecular characterization of high-risk stages II, III and IV melanoma patients, which is actually mandatory according to the European Society for Medical Oncology (ESMO) Clinical Practice Guidelines [11] and represents a fundamental step for tailored therapy. However, despite their important efficacies, primary and acquired resistance during treatment with BRAF plus MEK inhibitors still remain a significant challenge. In addition, targeted agents have shown several adverse events that may delay treatment and limit its effectiveness. The nature and incidence of BRAF and MEK inhibitor adverse oral events is actually not completely described; however, these complications may affect a patient’s quality of life or may require temporary or permanent cancer therapy termination. With this review, we set out to clarify the type, incidence and relative risk of adverse oral events in melanoma patients treated with the combination of BRAF and MEK inhibitors.

These adverse events should be carefully approached in a multidisciplinary team for an optimal treatment of patients with melanoma.

### 1.2. MAPK Pathway

Several mutated genes were identified as therapeutic targets and were involved in different molecular signaling pathways that are thought to be responsible for the carcinogenic process in melanoma cancer and its development, including the protein kinase B (AKT) pathway, mitogen-activated protein kinase (MAPK) pathway, cell-cycle regulation pathway, pigmentation-related pathway, p53 pathway, epigenetic factors and some others [12]. Mutations in the key signal components, including BRAF, NRAS, NF1 and KIT, are considered responsible for the hyper-activation of the MAPK pathway in melanoma cancer [13]. BRAF is a serine/threonine protein kinase, encoded on chromosome 7q34, that activates the MAP kinase/ERK signaling pathway. It is the family member most easily activated by Ras [14]. Almost 50% of melanomas show the activation of BRAF mutations. Among BRAF mutations in melanoma cancer, about 90% are sited at codon 600, and among these, over 90% are a single nucleotide mutation that results in the substitution of glutamic acid for valine (BRAFV600E: nucleotide 1799 T > A; codon GTG > GAG) [15]. The activation the of RAF protein inducts the phosphorylation of MAPK receptor kinase (MEK), which in turn phosphorylates extracellular signal-regulated kinase (ERK). The activation of the ERK protein activates cellular proliferation and mitochondrial proteins, which stimulate cell growth and inhibit cell apoptosis [16,17,18].

### 1.3. Current Standard of Care for Melanoma Cancer

Despite advances in melanoma treatment, the 5-year survival rate for patients with advanced melanoma remains poor.

The treatment of melanoma patients has been revolutionized by the introduction of targeted therapy and immunotherapy. Recently, these therapies have become standard care in the adjuvant and metastatic settings. Adjuvant therapy has been approved for stage III melanoma patients. Immunotherapy involves the administration of monoclonal antibodies, T-cells or immuno-stimulatory cytokines focused on priming the immune system and activating immune responses against tumor cells. In 1998, the first approved immunomodulatory for the treatment of advanced melanoma was IL-2; subsequently, interferon-α (IFN-α) has been the only approved agent for the treatment of high-risk cutaneous melanoma. This agent showed such a high rate of side effects that only 50% of patients were able to complete the entire year of therapy [19]. Ipilimumab approval was subsequently expanded for use in the adjuvant setting in 2015 after the phase III international EORTC 18071 trial comparing adjuvant high dose ipilimumab to a placebo in patients with completely resected stage III melanoma [20]. Despite these improvements in relapse-free survival (RFS) and overall survival (OS), ipilimumab has been shown to be associated with a high incidence of adverse events. However, given its toxicity and the introduction of new agents, adjuvant ipilimumab is no longer actually administered. Pembrolizumab and nivolumab are two antibodies that target the PD-1 checkpoint inhibitor. Both antibodies have demonstrated an increase in RFS in stage III melanoma patients [21,22]. Immune-related adverse events of anti-PD1 inhibitors include diarrhea, fatigue, anemia, nausea and decreased appetite with a grade 3–4 incidence of approximately 14% [23]. Recently, adjuvant pembrolizumab also improved RFS in high-risk stage II melanoma patients [24]. Based on these data, the European Medicines Agency (EMA) and Food and Drug Administration (FDA) approved pembrolizumab as an adjuvant treatment for stage II B or II C melanoma patients following complete resection.

Regarding target therapy, the treatment of melanoma was revolutionized with the discovery of the BRAF mutation in 2002. Vemurafenib was the first BRAF inhibitor approved by the FDA in 2011. Since then, the combination of BRAF and MEK inhibitors has been proven to be superior to single-agent BRAF inhibitors. In particular, the combination of the BRAF–MEK inhibitors dabrafenib and trametinib received regulatory approval after demonstrating significant improvements in RFS in stage III melanoma patients with BRAF V600E or V600K mutations [25]. In the current period of effective systemic therapies, the role of radiation therapy (RT) remains controversial [26]. RT looks promising and should be considered after regional lymph node dissection of macroscopic lymph node disease in patients with extracapsular extension; lymph node diameter >3 cm (neck or maxilla); or 4 cm (groin); or at least one involved lymph node in the parotid lymph node region, two in the neck and maxillary region or three in the groin region [27]. Radiation therapy can also be considered after the failure of previous regional lymph node dissection and after the resection of desmoplastic or other melanoma subtypes that show neurotropism. Moreover, patients with resectable metastases melanoma can be candidates to neoadjuvant treatment. Recently, several clinical trials have assessed the superiority of neoadjuvant approaches over standard surgery and subsequent adjuvant treatment [28,29,30,31].

The treatment of metastatic melanoma treatment should be discussed in interdisciplinary tumor boards with representation from multiple medical specialties. Anti PD-1 antibodies, either as monotherapy or in combination with anti-CTLA-4 ones, should be considered first-line treatment for all patients with unresectable metastatic melanoma and independent of tumor BRAF mutational status [32,33,34]. The association of nivolumab and ipilimumab has been shown to be superior in terms of progression-free survival (PFS) with ipilimumab or nivolumab as single agent drugs and is actually approved by the FDA and EMA [35,36,37]. In addition, in the presence of BRAFV600E/K mutation, combinations of BRAF and MEK inhibitors were able to show a significantly increased objective response rate, PFS and OS [38,39,40,41]. In the metastatic setting, there are now three combinations approved: vemurafenib and cobimetinib (V + C), dabrafenib and trametinib (D + T) and encorafenib and binimetinib (E + C) [26]. All these combination regimens showed long-term benefits as first-line treatment in patients who had unresectable or metastatic melanoma with a BRAF V600E or V600K mutation [42,43,44]. Direct head-to-head comparison of the available regimens is unlikely to be performed, but indirect side-by-side analysis of data from V + C, D + T and E + B compared to vemurafenib monotherapy [45] revealed comparable PFS and OS data. The availability of three approved BRAF-MEK inhibitor regimens gives multiple options of treatment for patients with stage IV BRAF-V600E/K mutant melanoma [46]. Finally, a small proportion of melanomas arising in sun-protected sites show mutations in cKIT. KIT inhibitors, most notably imatinib, have shown promising clinical activity in this subtype [47].

### 1.4. BRAF and MEK Inhibitors Adverse Events: Focus on Oral Side Effects

BRAF and MEK inhibitors are well tolerated, but some adverse events can occur because of paradoxical reactivation of MAPK signaling [48]. Inhibition of the MAPK pathway in keratinocytes can lead to inflammation, decreased keratinocyte cell migration and keratinocyte cell death, resulting in dermatologic side effects [49]. Skin toxicities are the most common adverse events associated with BRAF inhibitor, occurring in up to 57% of patients. Photosensitivity, rash, pruritis, dry skin, papilloma, alopecia, keratoacanthoma and squamocellular carcinoma (SCC) are the most common in BRAF inhibitor monotherapy, with photosensitivity resulting as primarily associated with vemurafenib [50]. Other adverse events that occur more frequently with combination therapy are fever (51%), chills (30%), fatigue (35%), diarrhea (24%), hypertension (22%) and vomiting (20%) [51].

Despite the efficacy of these drugs in the treatment of melanoma, one particular focus that can often be underestimated and that compromises the patient’s quality of life is the cutaneous and oral side effects of these drugs. Adverse reactions can vary from subject to subject and can have varying levels of severity, so a multidisciplinary approach is essential to prevent and manage possible complications, which are often responsible for lower adherence to therapy.

These drugs are endowed with specificity, acting on specific targets expressed exclusively by tumor cells and reducing the damage caused to healthy cells; the cells constituting the oral mucosa, however, having a high cell turnover, are, for this reason, more receptive to the action of these pharmacological agents and, therefore, are associated with a higher incidence of oral adverse effects. The latter are responsible for decreased quality of life and discomfort in terms of nutrition and less adherence to the therapy itself.

Associated with target therapy, one of the most adverse reactions found in the oral district is gingival hyperplasia, which is characterized by the presence of benign hyperkeratotic lesions with a warty appearance, and frequently finding localization at the level of the gingival mucosa, free gingiva and lips [52]. Although rare, a biopsy examination of the hyperkeratotic lesion is still indicated for doubtful cases of squamous cell carcinoma (SCC) associated with target therapy [53].

Other lesions, as described in the study conducted by Gençler et al. [54], can be squamo-proliferative: administration of vemurafenib as monotherapy has been observed to cause activation of the RAS pathway resulting in cutaneous and oral lesions such as squamous cell carcinoma, Keratoacanthoma and Acanthopapillomas.

Often associated with the administration of MEK inhibitors is Cheilitis Angularis, and it tends to occur six months after the start of therapy. It is a fissured lesion involving the corners of the mouth that become reddened and very painful, especially following lip movements, and it presents small cuts and abrasions until it ulcerates causing burning, dryness and suppuration [55].

Oral lichenoid reactions occur most frequently after the administration of multikinase inhibitors. They are closely related to the dose of the drug, and their treatment involves the use of corticosteroids and antihistamines [56].

Ulcerated, painful and atrophic lesions represent a clinical aspect of Mucositis, characterized by erythematous and reddened areas of the oral mucosa; often, the lesions have no dysplasia, only an infiltrate of inflammatory cells.

As outlined by Lueken et al. [57], pigmented lesions are associated with a melanocyte proliferation called Melanocytic Hyperplasia, found at the palatal level, mainly linked with tyrosine kinase inhibitors.

The relationship between concomitant BRAFi intake and periodontal disease is noteworthy; this condition is a chronic inflammatory process of the dental support tissues (periodontal ligament, gingiva, alveolar bone and radicular cementum) that results in the formation of periodontal pockets and resorption of bone support to the point of element loss. In this regard, treatment of periodontal disease with professional hygiene sessions, scaling, root planing and elimination of the inflammatory process will facilitate the healing process of these oral adverse events [58].

## 2. Materials and Methods

### 2.1. Design

This literature review follows the “Preferred Reporting Items for Systematic reviews and Meta-analyses” (PRISMA) checklist from 2009, a methodology including studies with qualitative, quantitative and mixed method designs to capture a greater latitude of the field.

### 2.2. Search Strategy

Literature searches were conducted by using four databases: Medline, Google Scholar, Embase and PubMed. Other relevant studies were identified from reference lists of systematic reviews and meta-analyses. Databases were used according to the inclusion criteria using filters to limit results to “full text” journal articles that were available at a price or free of cost, involving studies conducted in human participants aged at least 18 years old and being melanoma-specific. We did not apply any language restrictions in the searches. Retrospective, prospective and RCT research articles using a combination for “melanoma”, “Braf-Mek inhibitors”, “Targeted therapy” and “oral adverse events” were included. The first research was performed in April 2022, and no time limit was set. Because this is an emerging field of research, electronic searches were performed in all fields of the article. Two researchers (T.V., D.A.A.) executed 100% double title and abstract screening separately with inter-reviewer agreement. Studies that did not follow the inclusion criteria were excluded from full text review, and disagreement between researchers was resolved through discussion. Studies were subjected to full text review by two researchers independently (T.V., D.A.A.).

## 3. Results

### 3.1. Screening and Data Extraction

The first evaluation of the manually selected papers showed the necessity to establish some exclusion criteria because several articles were not focus-related to our matter of interest. A total of 30 studies were evaluated for title and abstract screening, and after removing duplicates, a total of 18 studies were judged to be eligible for full text review.

### 3.2. Data Synthesis

A descriptive and meta-analytical approach to data was considered appropriate to show our findings.

Among the most widely recognized side effects, we can mention SCARs (severe adverse skin reactions), which include the Stevens–Johnson Syndrome (SJS), Toxic Epidermal Necrolysis (TEN), drug rash with eosinophilia and systemic syndrome (DRESS), Acute Generalized Exanthematous Pustulosis (AGEP) and Generalized Bullous Fixed Eruption (GBFE) [59,60]; these occur in 20–30% of cases [61].

Torres-Navarro et al., in their study, analyzed the effects produced by some of these drugs; for example, encorafenib, binimetinib, dabrafenib, trametinib, vemurafenib and cobimetinib.

No skin reactions were found for cobimetinib as a monotherapy; instead, trametinib, encorafenib or binimetinib (alone or associated with dabrafenib), were associated in two cases of DRESS [62,63].

Most of the side effects are from the treatment with vemurafenib in association with cobimetinib, but the association in cases of health-threatening SCARs has been confirmed to be between dabrafenib and trametinib [25,64] instead; in contrast, SCARs are less frequent for the encorafenib–binimetinib association [39].

Fractures and osteopenia are serious complications found in two patients treated for a long time with MEKi. In the research of Dumas et al. [65], especially, the first patient with melanoma and under treatment with “pimesertib” for 6 years developed a clinical picture characterized by osteopenia, sacrum fracture, increased alkaline phosphatase levels and vitamin D deficiency.

In the second melanoma patient treated for about 6 months with BRAFi/MEKi combination, a pelvic bone fracture was found at the level of the left acetabulum [66].

Among the adverse effects of these drugs are oral adverse events. In this regard, the study by Dika et al. evaluated these effects in patients affected by melanoma under treatment with BRAFi/MEKi and tyrosine kinase inhibitors and in patients treated with CTLA and PD1 inhibitors [67].

The first group of patients had a clinical picture characterized by gingival hyperplasia (multiple hyperkeratotic lesions at palatal, gingival, tongue and lip levels) [68,69], blue-brown-black pigmentations at the palatal level [70,71] and proliferative lesions such as keratoacanthoma (KA) and squamous cell carcinoma (SCC) [72,73,74].

In contrast, in the second group, the adverse effects are lichenoid reactions with possible risk of neoplastic evolution [75,76], bullous pemphigoid and erythema multiforme [67] and xerostomia with subsequent dysgeusia.

Additional adverse reactions, such as hypertension, diarrhea, fever, skin rash, hemorrhage, mucositis and oral ulceration [71], were relevant from the research of Lyne et al., who studied the adverse effects of the drug Apatinib in refractory or relapsed melanomas.

In addition to these adverse reactions, there is also a case of Actinomycosis revealed by ulcerations of the palate and gingiva; in fact, the study by Dessirier et al. examined a case of melanoma treated with BRAFi, which presented itself with oral, bleeding and hyperalgesic ulcerations.

The histologic examination showed the presence of a filamentous appearance typical of Actinomycosis, thus requiring treatment with an antibiotic (penicillin G) [77].

Hyperkeratosis, hyperplasia, inflammation and gingival ulceration, on the other side, represent the toxicity profile of a melanoma case, undergoing treatment with vemurafenib for the duration of 2 years [58].

The patient, subjected to a treatment plan based on scaling, root planing, periodontal tooth extractions and preservation of a high level of oral hygiene resulted in resolution of gingival abnormalities.

Damsin’s study found endobuccal toxicities in a patient with colorectal adenocarcinoma treated with target therapy; these include aphthoid lesions that tend towards confluence and mucositis.

The main problem is related to overinfection and nutritional disorders of the patient; oral hygiene, rinses with antiseptic mouthwashes and specific nystatin–hydrocortisone–lidocaine solutions are essential [78].

Hyperkeratotic lesions are the most prevalent side effects for vemurafenib and dabrafenib when used in monotherapy. These include keratosis-like rashes, warty papillomas, and tumor-like lesions, such as keratoacanthoma or squamous cell carcinoma.

Oral toxicities have also been described, such as hyperkeratotic lesions on the marginal gingiva, palate, tongue and labial mucosa and vemurafenib-induced Gingival Hyperplasia.

A case of squamous cell carcinoma on vemurafenib-induced lesions on the labial mucosa has also been reported [79,80]. Table 1 reports a resume of the findings.

## 4. Discussion

The nature and incidence of adverse oral events associated with BRAF and MEK inhibitor therapy are incompletely described. However, these complications may affect a patient’s quality of life or may require temporary or permanent cancer therapy termination. With this review, we set out to clarify the type, incidence and relative risk of adverse oral events in patients with melanoma who are being treated with a combination of BRAF and MEK inhibitors.

These adverse events should be carefully approached in a multidisciplinary team for an optimal treatment of patients with melanoma.

Fundamental is the interdisciplinary approach to treating the patient from every point of view, based on dialogue and collaboration among the various specialists.

This makes it possible to improve the response to the treatments performed, allowing for the effective management of the eventual response of the disease, giving the possibility to take action during all stages of the pathology and taking into account histological and clinical factors.

It is also important to plan adequate monitoring of the efficiency of the professional team, to define the responsibilities of the different clinicians and to use a set of indicators of effectiveness.

The collaboration of oncologists, dermatologists, anatomo-pathologists, nuclear physicians, radiologists, plastic surgeons, dentists, psychologists and the nursing staff allows for framing the patient at the center of a path of care, support and rehabilitation.

To optimize patient management, Claveau et al. emphasize the implementation of a regional multidisciplinary team in order to identify, in the same region, specialists, means of communication of team members, possible obstacles and treatment strategies [81].

The importance of the multidisciplinary team approach (MDT) in the management of the melanoma patient has been reviewed by the study by Cornelius Lynn et al.; the team allows for optimization of care, improved diagnostic accuracy and timely management of side effects [84].

The perspective of the dermatologist who is part of the MDT is to be an active participant in every phase of patient management; the role of the surgeon who is responsible for evaluating and choosing the most appropriate surgical technique is also important.

Instead, it is the responsibility of the oncologist to review the exact clinical and histologic staging of the tumor, discuss treatment options and provide the patient’s educational materials [82].

A Diagnostic Therapeutic Care Pathway (PDTA) has been formalized for melanoma; it provides for cancer patient care, improved care and reduced time for diagnosis, treatment, staging and follow-up.

This pathway provides a reduction in the economic impact on patient management, a prolongation of the survival rate, a high standard of the quality of medical and surgical treatment, an appropriate and efficient treatment according to the individuality of each patient and, lastly, it promotes access to advanced therapies provided by national and international trials in order to limit the movement of patients to other centers in the nation.

Treatment with BRAFi/MEKi for a melanoma is not exempt from side effects, and we may experience adverse reactions especially in the oral cavity. These adverse effects affect patients’ lives by causing functional, aesthetic and masticatory alteration of the stomatognathic apparatus.

In this regard, it is of paramount importance to establish an excellent dentist–patient relationship in order to tailor a treatment plan according to the type and severity of side effects [83].

Dental management will therefore be based on accurate medical history; identifying hereditary conditions; personal physiological for assessing habits, allergies, socio–economic factors, medications taken, alcohol use, smoking and lifestyle; and personal pathological for assessing all past medical interventions, any complications and current issues related to BRAFi/MEKi intake.

The next step will be the objective examination, both extraoral and intraoral, that allows us to examine the current status of mucous membranes; changes in color, shape, texture, function of oral structures; evaluation of signs; and symptoms pathognomonic of pathological processes.

To complete the diagnostic process, the realization of the Clinical and Periodontal Record will be required; then, it will be necessary to evaluate Radiographic Examinations based on Rx Orthopantomography, Rx Endoral and ConeBeamCT to assess radiopacity and radiotransparencies to expand the objective examination. Lastly, laboratory tests will be conducted to allow for the evaluation of blood parameters, such as blood count, leukocyte formula, blood glucose, glycosylated hemoglobin and indices of systemic inflammation.

The aforementioned requirements will enable the creation of the treatment plan that will provide for the resolution of oral problems; it will be based on professional oral hygiene, treatment of incongruous fillings, devitalization of non-viable dental elements, remedies to prevent systemic contamination, prosthetic restorations and the use of devices for controlled release of topical antibiotics or chlorhexidine solutions.

It is crucial to recognize the first symptoms of chemotherapy-induced mucositis in order to avoid further complications. The inflammatory process in the mucous membranes causes a loss of mucosal function with the risk of overinfection.

Currently, there are no guidelines for the treatment of chemotherapy-induced mucositis; the choice falls between pharmacological and non-pharmacological therapies. There is plenty of scientific evidence affirming the efficacy of compounds, such as the hydroglyceric extract of propolis and grapefruit and another active ingredient, ectoin.

The latter has the function of moisturizing the mucous membranes, providing immediate relief and promoting mucosal healing.

## 5. Conclusions

This review addresses melanoma and its therapy, with a focus on targeted BRAFi/MEKi treatment. Patient involvement and multidisciplinary management are essential for effective treatment. Collaboration between oncologists and dentists is crucial for early diagnosis, investigations and regular check-ups to prevent and treat oral complications, thus improving patients’ quality of life and adherence to treatment.

## Figures and Tables

**Table 1:** Resuming table of the result section. NA: not applicable.

Authors	Target Terapy	Number of Patients	Toxicity	Treatment
Rouleau JC et al. [52]	NA	50% of hospitalized patients	Stevens–Johnson Syndrome (SJS) (Erosive stomatitis)	Appropriate diet, antibacterial treatment, plasmapheresis
Duong TA et al. [60]	NA	NA	Drug rash with eosinophilia and systemic syndrome (DRESS)	Oral corticosteroids, antipyretics, patch testing at month 6
	NA		Oral mucous membrane involvement	Topical steroids, antipyretics, patch testing after 6 weeks
Lamiaux et al. [62]	Vemurafenib associated with cobimetinib	one case	Stevens–Johnson syndrome	Corticotherapy (case by case)
	Vemurafenib associated with cobimetinib	four cases	Drug rash with eosinophilia and systemic symptoms (DRESS)	Corticotherapy (case by case)
G.V. Long et al. [65]	Dabrafenib plus trametinib	438 patients	Keratoacanthoma was reported in eight patients	NA
Dummer R et al. [66]	Encorafenib or vemurafenib	52 patients	Dysgeusia	NA
Robert C. et al. [67]	Dabrafenib plus trametinib	699 patients	Hyperkeratosis in 15 patients	NA
	Vemurafenib		Hyperkeratosis in 88 patients	NA
Dumas et al. [68]	Dabrafenib with trametinib	two patients	Fractures with osteopenia	Calcium, vitamin D, phosphate supplements or calcitriol
Dika et al. [70]	NA	NA	Gingival hyperplasia	Follow-up
	Nivolumab	NA	Lichenoid reactions	Topical corticosteroids
	Anti-PD-1 treatment	NA	Immunobullous Reactions	NA
	Nivolumab or pembrolizumab	NA	Xerostomia	Accurate oral hygiene, dietary advice
	Imatinib	NA	Pigmentation disorders of the palate	Clinical and dermoscopic follow-up
	Vemurafenib	NA	Squamoproliferative lesions	NA
	Bevacizumab, sorafenib and sunitinib	NA	Benign migratory glossitis	NA
Lyne et al. [74]	Imatinib	one case	Oral mucosal pigmentation	NA
Su et al. [75]	Vemurafenib	NA	Keratocantoma	NA
Dika et al. [76]	Vemurafenib	Seven patients	Squamo-proliferative epithelial neoplasms	NA
Vigarios et al. [77]	Vemurafenib, dabrafenib	NA	Hyperkeratotic lesions (linea alba, hard palate, gingiva…), hyperplasia of gum, secondary squamous cell carcinoma	NA
Carrozzo et al. [78]	NA	NA	Oral lichen planus	NA
Fitzpatrick et al. [79]	NA	NA	Oral lichen planus Oral lichenoid lesions	NA
Dessirier et al. [80]	Dabrafenib	one case	Actynomicosis	Antibiotics
Shephard et al. [81]	Vemurafenib	one case	Hyperkeratosis, hyper-plasia, ulceration and inflammation of the gingivae	Periodontal treatment
Peterson et al. [82]	NA	NA	NA	NA
Vigarios et al. [83]	Dabrafenib with vemurafenib	NA	Hyperkeratotic lesions	NA
Mangold et al. [53]	Vemurafenib	one case	Gingival hyperplasia	NA
Gençler et al. [55]	Vemurafenib, dabrafenib	NA	Squamo-proliferative lesions	NA
Balagula et al. [56]	Selumetinib	8	Cheilitis angularis	NA
Livingstone et al. [57]	Multikinase inhibitors (imatinib, dasatinib e nilotinib)	NA	Oral lichenoid reactions, mucositis and oral ulcerations	Corticosteroids, antihistamines
Lueken et al. [58]	Tyrosine kinase inhibitors	one case	Pigmented lesions	Follow-up

## Data Availability

Not applicable.
